# Progress, Opportunities, and Challenges of Magneto-Plasmonic Nanoparticles under Remote Magnetic and Light Stimulation for Brain-Tissue and Cellular Regeneration

**DOI:** 10.3390/nano12132242

**Published:** 2022-06-29

**Authors:** Muzhaozi Yuan, Mackenzie Caitlin Harnett, Tian-Hao Yan, Elias Georgas, Yi-Xian Qin, Hong-Cai Zhou, Ya Wang

**Affiliations:** 1 J. Mike Walker ‘66 Department of Mechanical Engineering, Texas A&M University, College Station, TX 77843, USA; mh2255@tamu.edu; 2 Department of Chemistry, Texas A&M University, College Station, TX 77843, USA; thyan426@tamu.edu (T.-H.Y.); zhou@chem.tamu.edu (H.-C.Z.); 3 Department of Biomedical Engineering, State University of New York, Stony Brook, NY 11794-5281, USA; elias.georgas@stonybrook.edu; 4 Department of Biomedical Engineering, Texas A&M University, College Station, TX 77843, USA; 5 Department of Electrical and Computer Engineering, Texas A&M University, College Station, TX 77843, USA

**Keywords:** magneto-plasmonic nanoparticles, neurodegenerative disease, magnetic field, light stimulation, controlled and sustained drug release, brain organoid

## Abstract

Finding curable therapies for neurodegenerative disease (ND) is still a worldwide medical and clinical challenge. Recently, investigations have been made into the development of novel therapeutic techniques, and examples include the remote stimulation of nanocarriers to deliver neuroprotective drugs, genes, growth factors, and antibodies using a magnetic field and/or low-power lights. Among these potential nanocarriers, magneto-plasmonic nanoparticles possess obvious advantages, such as the functional restoration of ND models, due to their unique nanostructure and physiochemical properties. In this review, we provide an overview of the latest advances in magneto-plasmonic nanoparticles, and the associated therapeutic approaches to repair and restore brain tissues. We have reviewed their potential as smart nanocarriers, including their unique responsivity under remote magnetic and light stimulation for the controlled and sustained drug delivery for reversing neurodegenerations, as well as the utilization of brain organoids in studying the interaction between NPs and neuronal tissue. This review aims to provide a comprehensive summary of the current progress, opportunities, and challenges of using these smart nanocarriers for programmable therapeutics to treat ND, and predict the mechanism and future directions.

## 1. Introduction

**Figure 1:**
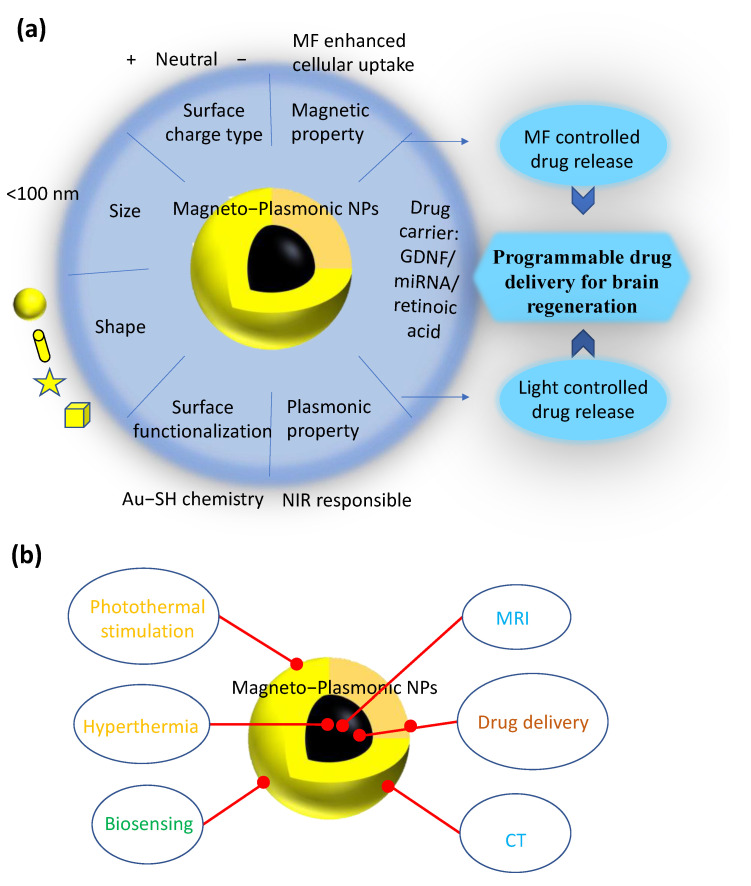
Schematic of (**a**) magneto-plasmonic nanoparticle (NP) properties and their application for programmable drug delivery; (**b**) applications of magneto-plasmonic NPs for brain-tissue and cellular regeneration.

As one of the major threats to human health, neurodegenerative diseases (NDs) have an increasing prevalence in the elderly population due to the extended lifespan nowadays [1]. One of the promising therapeutic strategies for ND development in recent years is the use of nanocarriers to deliver neuroprotective and neurorestorative drugs [2], neurotrophic factors [3], and genes [4]. Among the potential nanocarriers, magneto-plasmonic nanoparticles (NPs) possess strong potential to overcome the current therapeutic limitation of ND. On the one hand, as shown in Figure 1a, their magnetic nature enables them to be externally directed by a static MF, to generate heat under alternating MFs, to cross the blood–brain barrier (BBB) under MF stimulation, and to regulate mechanosensitive ion channels [5]. On the other hand, their plasmonic nature enables the photothermal conversion capacity and the light-triggered neural regulation of temperature-sensitive ion channels [6]. Therefore, magneto-plasmonic NPs can serve as an outstanding platform to realize targeted drug delivery and external-stimuli-controlled drug release. In addition, they are capable of passing through the BBB because of their small size (1–100 nm) and ability to enable facile surface modification. The surface chemistry of magneto-plasmonic NPs also enables tunable surface functionalizations, which suggests that they could be superior drug carriers of various therapeutic agents to cross the BBB. These unique properties and biological functions allow for the wide usage of magneto-plasmonic NPs for brain-tissue and cellular regeneration, including photothermal stimulation, the biosensing of proteins/molecules, hyperthermia, bioimaging (magnetic resonance imaging (MRI), computed tomography (CT)), and drug delivery (Figure 1b).

Previous literature has summarized the recent advances in the use of various types of nanomaterials as carriers for the brain drug delivery of Parkinson’s disease (PD) [7], and the related surface phenomena [8], as well as the functions to cross the BBB [9,10]. Torres-Ortega et al. reviewed the current micro- and nano-based approaches, and especially for the administration of therapeutic agents [11]. Falconieri et al. summarized the work of using magnetic NPs to promote neuroregeneration till 2019 [5], while Paviolo et al. summarized the special use of gold NPs to modulate neuronal activities and behavior till 2017 [6]. One recent review summarized the advances in magnetic gold NPs, which mainly focus on synthesis approaches, surface-modification methods, and the related biomedical applications [12]. However, there is still a lack of sufficient and up-to-date overviews of the latest advances in magneto-plasmonic NPs that summarize the impact of their unique magneto-plasmonic properties on cellular interactions, their BBB-crossing behavior, and their drug-delivery strategy. There is also a lack of reviews on combining magneto-plasmonic NPs with different external-stimulus types for the treatment of ND, and discussion on the translational potential of these NPs to develop programmable disease-modifying therapy in ND. 

Therefore, in this review, we intend to summarize these crucial aspects and provide an up-to-date comprehensive review of the unique properties of magneto-plasmonic NPs, as well as of the progress and challenges of technologies/strategies based on the combined effect of NPs and various external stimuli for neuroregeneration. Importantly, we focus on providing summaries on applying external stimuli to modulate magneto-plasmonic NP–cell interactions for cellular uptake and BBB crossing, and to control drug release for the treatment of ND. We also intend to discuss the application of brain organoids to analyze the impact of NPs on neuronal activities in a 3D biomimicking environment, and we propose the possible opportunities for developing programmable disease-modifying treatment.

## 2. Types and Properties of Magneto-Plasmonic NPs

### 2.1. Superparamagnetic Iron Oxide–Gold (SPIO–Au) NPs

Multifunctional magneto-plasmonic NPs, which combine superparamagnetic iron oxide (SPIO) with gold (Au), are becoming more and more attractive for the development of neuroregeneration therapeutics. One of the common structures is the core–shell structure, such as SPIO–Au (SPIO as core and Au as shell) and Au–SPIO (Au as core and SPIO as shell). On the one hand, Au–SPIO core–shell NPs have been less investigated due to the challenge in synthesis to encapsulate the Au core inside the SPIO shell. Elena et al. reported the synthesis of Au–SPIO core–shell NPs and found that the deposition of SPIO on the Au core could significantly affect the plasmonic properties of Au NPs, such as by reducing the localized surface plasmon resonance (LSPR) intensity [13]. On the other hand, SPIO–Au core–shell NPs are the most widely developed magneto-plasmonic structures, and they have multi-benefits from SPIO and Au by protecting the iron oxide core using the Au shell and providing facile surface functionality with biomolecules through the well-known Au–thiol linkage [2]. Such SPIO–Au NPs have been proven as promising candidates as dual-mode contrast agents for CT and MRI because of the X-ray attenuation effect by the Au shell and the magnetic property of the SPIO core [14]. Banerjee et al. [15] theoretically and experimentally proved that the surface coating of SPIO with Au can enhance the magnetization by six times, which is attributed to the interfacial effect and related to the formation of a large magnetic moment between SPIO and Au. Recently, we have shown that SPIO–Au core–shell NPs functionalized with nerve growth factor (NGF) can promote in vitro neuronal growth and differentiation under the stimulation of light [16] and dynamic MF [17]. We have further proven that SPIO–Au NPs in combination with static MF stimulation are capable of regulating calcium fluxes by modulating L-type voltage-gated ion-channel activities, which, in turn, influences the downstream apoptosis signaling pathway in primary midbrain neurons [18]. Noticeably, during this process, the existence of an MF enhances the cellular uptake of SPIO–Au NPs. In addition, the multifunctionalities of SPIO–Au NPs make them optimal candidates for biosensing. Lee et al. developed a hybrid Au–SPIO NP–carbon nanotube nanoplatform for the biosensing of virus DNA [19]. In their work, the existence of Au could help the conjugation of probe DNA with the platform via the thiol-group interaction. The existence of SPIO can further help the alignment of nanoplatforms on electrodes guided by the MF. Due to the unique light/magnetic-force-responsive properties of Au–SPIO multifunctional NPs, they also have strong potential for external-stimuli-controlled drug delivery and programmable neuroregenerative therapy, which will be reviewed and summarized in Section 6.

### 2.2. SPIO–Hollow Gold Nanoshell (HGNS) NPs

**Figure 2:**
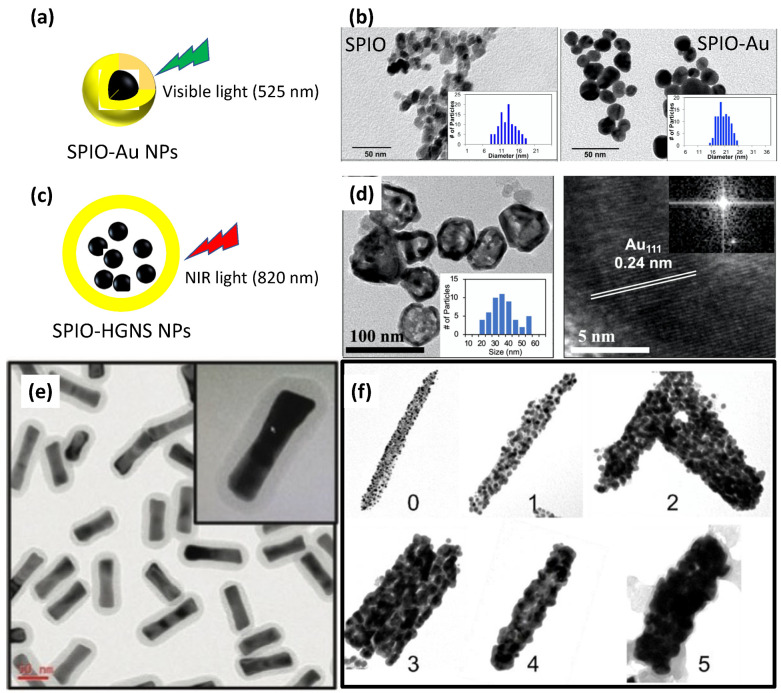
(**a**) Schematic of SPIO–Au-NP structure enabling 525 nm light stimulation. (**b**) TEM images of SPIO and SPIO–Au NPs (Copyright 2018 by Elsevier); (**c**) schematic of SPIO–HGNS-NP structure enabling 820 nm NIR light stimulation; (**d**) TEM images of SPIO–HGNS NPs and Au_111_ lattice (Copyright 2022 by Springer). (**e**) TEM images of PEG-conjugated and silica-coated gold nanorods (Copyright 2017 by Wiley). (**f**) TEM images of SPIO–Au nanorods at an increasing gold/SPIO seed ratio (Copyright 2022 by American Chemical Society).

Although SPIO–Au NPs have shown promising therapeutic potential for neuroregenerative therapy, their LSPR peak, located in the visible light region, limit their applications. For example, the LSPR peak of SPIO–Au core–shell NPs synthesized using the hydroxylamine seeding method [15] (Figure 2a,b) is located at 500–600 nm, which has a very short tissue-penetration depth and restricts the in vivo brain applications for neuron regeneration requiring a strong tissue-penetration ability. To turn the LSPR peak of SPIO–Au NPs from the visible region to the near-infrared (NIR) region, which has a longer tissue-penetration depth, one way is to form an additional interlayer at the surface of the SPIO, and then form an Au coating, which has a relatively large outer diameter and smaller Au-shell thickness, and thereby realize the LSPR tuning from the visible to NIR region [20]. Another way is to trap SPIO NPs inside HGNSs, which have LSPR peaks in the NIR region [21]. However, these methods lack control over the LSPR peak to a specific wavelength in the NIR region. We have recently developed SPIO–HGNS structures with tunable LSPR peaks from 820 nm to 945 nm using a silver-template-enabled two-step galvanic replacement reaction, as shown in Figure 2c,d [22]. It is shown that, with a larger inner diameter of the SPIO–HGNS, the LSPR peaks are red-shifted. This relationship between the size and LSPR properties is also revealed by the simulation of the absorption cross-sections of SPIO–HGNS NPs of similar sizes, which suggests a way of tuning the LSPR properties by precisely controlling the size of the HGNS [22]. 

### 2.3. Magnetic Au Nanorods

So far, studies of nanomaterials for brain diseases are mainly focused on NPs that have spherical shapes because of the easiness of the fabrication process. However, recent reports show that nanorods can penetrate the BBB more efficiently than spherical NPs [23]. Lee et al. compared the ability of rabies virus glycoprotein (RVG) 29-coated Au nanorods (length: 117.7 nm, width: 50.3 nm, as shown in Figure 2e) with their spherical controls (73.9 nm) [24]. They found that, for up to 24 h check points, the Au nanorod exhibited higher brain localization than the spherical groups. This difference in the BBB-passage ability is possibly due to the curvature difference between nanorods and spherical NPs. The smaller curvature of nanorods can enhance their binding to the surface of endothelial cells [23]. In addition, Au nanorods possess two LSPR peaks in transverse and longitudinal directions, which are later tunable from the visible light region to the NIR region. This feature makes them particularly attractive in many biomedical applications. With the combination of the Au nanorod shell and the magnetic core, as magnetic Au nanorods, their application can be further extended due to the benefits, such as multifunctionality, highly tunable magnetic and optical properties, and shape-enhanced BBB crossing. The configuration and fabrication of this nanomaterial was recently reported by Rincon-Iglesias et al. [25]. They first synthesized Fe_3_O_4_ nanorods as the core with controllable length and width by changing the capping-agent amount. Then, the Au nanocluster (below 3 nm) as seeds were deposited onto the surface of the Fe_3_O_4_ nanorods to form a complete Au shell by the growing process, as shown in Figure 2f. These multifunctional magnetic Au nanorods showed a high magnetization rate of 83.1 A m^2^/kg, and an LSPR peak located at 613 nm, which suggest their potential for magnetic hyperthermia and photothermia. However, the light absorbance in the NIR region is still restricted, and the method of forming the Au coating makes it hard to realize the thickness control. There is a particular need to modify the current method, or develop a new synthesis method, to fabricate magnetic Au nanorods with an LSPR peak that can be tuned from the visible region to the NIR region. Thus, a higher photothermal conversion efficiency can be obtained under NIR light irradiation. 

### 2.4. Material-Orientation Dependence of Magneto-Plasmonic Properties

The magneto-plasmonic properties of NPs are highly dependent on their anisotropic orientation. Traditional SPIO NPs usually have small sizes (below 20 nm). However, at this size range, their magnetic moments are small, which limits their magnetic response. To overcome this limitation, iron oxide NPs were developed in different shape anisotropies, such as nanochains, nanobundles, and nanorods [26]. Iron oxide nanorods with excellent superparamagnetic behavior have been studied as drug-delivery vesicles and contrast agents, with a high performance in MRI imaging [27]. It is also reported that magnetic hyperthermia can be modulated by using SPIO NPs of different shapes, such as spherical, rod-like, and cuboidal shapes [28]. Among them, the cuboidal shape exhibited the maximum specific-absorption rate. Plasmonic properties are also affected by the anisotropic structures, as LSPR is highly affected by the size and morphology of plasmonic nanostructures. Anisotropic Au nanomaterials, such as nanorods, nanocubes, nanoflowers, etc., have much higher reflective index sensitivities and tunable SPR peaks, which can reach the NIR region by modulating the aspect ratio [29]. Recently, Feng et al. reported a method to enable the vertical self-alignment of plasmonic nanorods to the colloidal substrate. This method suggests the possibility of designing magneto-plasmonic NPs with tunable plasmonic excitation by the MF-assisted control of the anisotropic orientation [30].

## 3. Toxicity and Cellular Uptake of Magneto-Plasmonic NPs

Uncoated magnetic NPs, such as SPIO, cause multiple adverse effects, such as increasing the expression of inflammation-related genes, and decreasing the level of intracellular reduced glutathione [31]. Coating the surfaces of SPIO NPs with gold may dramatically reduce their toxicity, as well as their cellular uptake. Our previous work demonstrated that citrate anion-capped or NGF-functionalized SPIO–Au core–shell NPs at 21 nm have excellent in vitro cellular viability in the MC-3T3 cell line [32] (viability > 93%, 7 days) and PC-12 cell line [17] (viability > 96%, 5 days) at 10–80 ug/mL dosage levels, without affecting the cell morphology. The cell proliferation rates of both cell lines are also enhanced by SPIO–Au NPs.

Bulk Au is inherently nontoxic; however, there might be toxicity in Au NPs due to the effect of surface-functionalizing ligands and their shape and size. First of all, the toxicity can be affected by the surface-functionalization type and charge of the NPs. For example, it was found that citrate- and biotin-functionalized Au NPs are not toxic when used up to a concentration of 250 mM, whereas hexadecyltrimethylammonium bromide (CTAB)-coated Au NPs are toxic to K562 cells at 0.05 mM [33]. Schaeublin et al. reported that neutral Au NPs and charged Au NPs induced cell death through necrosis and apoptosis, respectively [34]. It was further revealed by Goodman et al. that anionic (carboxylate ligand) Au NPs are nontoxic, while cationic (ammonium ligand) Au NPs show moderate toxicity [35]. This toxicity is due to the interactions between the cationic ligand and the cell membrane, which are mediated by the attractive effect between the negatively charged bilayer and cationic NPs. 

The cellular-uptake efficiency of magneto-plasmonic NPs can also be affected by the surface-functionalization type. A higher cellular-uptake rate is usually observed for cationic NPs, which is facilitated by the attractive electrostatic interaction between NPs and negatively charged cell membranes [36]. The surface-charge magnitude is another factor that also affects the cellular-uptake rate: a higher magnitude leads to a higher uptake rate, regardless of the surface-charge type (positive or negative) [37]. 

Secondly, the shape of NPs is also one of the critical parameters that determine their toxicity and cellular-uptake efficiency. Carnovale et al. revealed that CTAB-coated spherical and prismatic Au NPs have cytotoxicity, whereas CTAB-coated Au NPs in rod and cube shapes are tolerated by cells, and are more biocompatible at the same concentration [38]. They suggested that the higher toxicity of spherical Au NPs may come from their much higher cellular-uptake rate compared with Au NPs in rod and cube shapes. Xie et al. reported that the cellular-uptake efficiency is affected by the shapes of Au NPs in the following order: stars < rods < triangles, which is possibly due to the different endocytosis pathways and different portions of cellular-uptake pathways by different shapes [39]. For example, the cellular uptake of Au NPs in triangle shapes was dominated by clathrin-mediated pathways, whereas the cellular uptake of Au NPs in rod shapes was dominated by both the clathrin- and caveolae-mediated pathways, which may result in a lower cellular-uptake efficiency.

Thirdly, the size of Au NPs also affects their toxicity and cellular-uptake efficiency. It was shown that 20 nm Au NPs administered intravenously could pass through the BBB without inducing cytotoxicity [40]. In vivo evaluations revealed that citrate anion-capped Au NPs at 3, 5, 50, and 100 nm did not show harmful effects on mice at a dose of 8 mg/kg/week [41]. However, polyethylene glycol (PEG)-coated Au NPs showed increased toxicity in Hela cells, with a reduced size from 61.2 nm to 6.2 nm [42], which was due to the different oxidative stress induced by different sizes [43]. Pan et al. also explored the toxicity of Au NPs from 0.8 to 15 nm [44]. They found that Au NPs at the size of 1.4 nm caused rapid cell death through necrosis compared with other sizes. The size dependency of the cellular uptake of Au NPs is observed in different cell lines. It is reported that Au NPs at 50 nm showed a much higher uptake efficiency than Au NPs at 13 nm in human primary glioblastoma cells [45]. In Hela cells, citrate-capped Au NPs showed the maximum uptake rate at a size of 50 nm, compared with 14 and 74 nm [46], while, in breast-cancer cells, the ultrasmall Au NPs at 2 nm showed the maximum uptake in sizes ranging from 2 to 15 nm [47].

**Figure 3:**
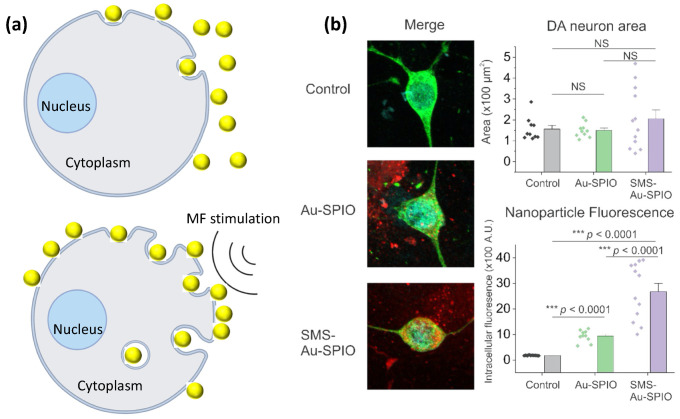
MF stimulation on enhancing cellular uptake (**a**) Schematic illustration of how MF stimulation enhances the cellular uptake of magnetic NPs by strengthening the sedimentation process of NPs onto the cell membrane; (**b**) confocal-microscope images showing the enhanced cellular uptake of SPIO–Au NPs into midbrain dopaminergic neurons under the stimulation of static MF. Grey bar represents Control group; Green bar represents Au-SPIO group; Purple bar represents SMS-Au-SPIO group. *** *p* < 0.001 was statistically considered significant difference. NS = not significant. Copyright 2022 by ACS. (Created with BioRender.com on 1 April 2022).

Last but not least, another critical factor that can affect the cellular-uptake efficiency of magneto-plasmonic NPs is the external stimulation, such as MFs, as summarized in Table 1. It is reported that an external static MF can enhance the cellular uptake of silica-coated SPIO NPs in a magnetic-intensity-dependent manner [48]. This enhancement is because of the strengthened sedimentation process of SPIO NPs onto the surface of the cellular membrane by magnetic force, as illustrated in Figure 3a. Venugopal et al. reported that the cellular uptake of SPIO–Au NPs into tumor cells can be enhanced by a static MF [49]. This enhancement by MF is due to the magnetization of SPIO–Au NPs and the generation of an attractive force, which enhances the translocation of SPIO–Au NPs through the cellular membrane. Our recent work also revealed that the static MF can enhance the uptake of negatively charged SPIO–Au NPs into midbrain dopaminergic neurons, as shown in Figure 3b [18]. A theoretical model was developed by Li et al. to study how MFs and magnetic NPs affect the cellular-endocytosis process [50]. They found that the MF effect is size-dependent, and that there are a maximum radius (R_max_) and a minimum radius (R_min_) of magnetic NPs that are thresholds for different endocytosis mechanisms. When the radius of magnetic NPs is R < R_min_, the magnetic NPs cannot enter the cells through receptor-mediated endocytosis due to the high membrane-deformation cost and weak magnetic force by small magnetic NPs; when R_min_ < R < R_max_, the receptor-mediated endocytosis is favorable due to the reduced membrane-deformation cost and the higher binding energy and magnetic force. When R > R_max_, the cellular internalization pathway is mainly mediated by the MF because the MF is strong enough to overcome the membrane-deformation cost. 

## 4. External-Stimuli-Assisted BBB Passage of Magneto-Plasmonic NPs

The existence of the BBB, as a unique structure that protects the brain, significantly limits the penetration of therapeutic agents into the targeted brain area. Magneto-plasmonic NPs have been developed to penetrate the BBB for brain-neuron regeneration, and most of them exhibit a size dependency on the BBB-passage efficiency. For example, in an in vitro model of the BBB, barbiturate-coated Au NPs at the size of 70 nm showed the maximum accumulation in the brain cells, compared with Au NPs at sizes of 20, 50, and 110 nm [57]. Others found that insulin-coated Au NPs showed the highest accumulation of gold in the brain at the size of 20 nm in an in vivo study [58]. Therefore, the surface-functionalization type and size may dramatically affect the BBB passage of magneto-plasmonic NPs. 

**Figure 4:**
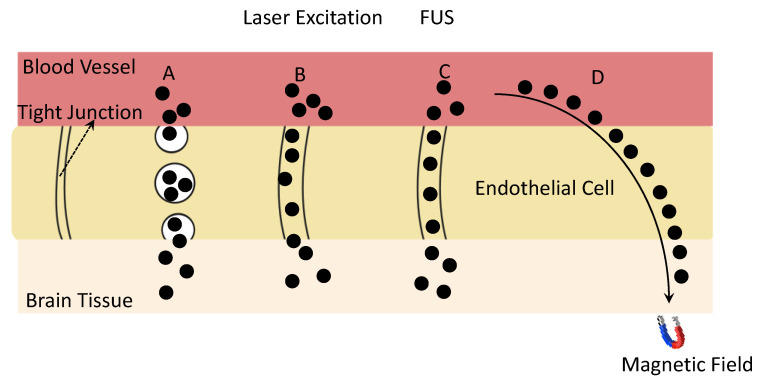
Schematic illustration of BBB-crossing pathways of magneto-plasmonic NPs assisted by surface functionalization and different external stimulations: (**A**) receptor-mediated transcytosis enhances the uptake of NPs functionalized with specific ligands; (**B**) paracellular transport of NPs by disrupting the tight-junction system under the irradiation of laser light; (**C**) paracellular transport of NPs by disrupting the tight-junction system under the exposure to FUS; (**D**) targeted passage of NPs through BBB directed by MF.

BBB-targeting agents can be functionalized onto magneto-plasmonic NPs to further enhance the BBB-passage efficiency of NPs by enhancing the receptor-mediated transcytosis, as shown in Figure 4. A study by Khonghow et al. shows that Au NPs coated with brain-targeted exosome exhibited stronger binding with brain cells and had an improved BBB passage in an in vitro BBB model composed of endothelial cells and astrocyte-like cells [59]. The in vivo study also showed a higher brain uptake of Au NPs coated with brain-targeted exosome. Insulin is another targeting agent that is widely used to enhance the BBB passage of NPs. Betzer et al. reported that the conjugation of Au NPs with insulin can enhance their brain accumulation by about five times because of the receptor-mediated-endocytosis process by insulin [60].

The shape of NPs is another critical factor that affects BBB transport. The NPs with a rod-like morphology gain a much higher BBB-passage rate compared with those of a spherical shape due to the stronger cellular interaction by the higher aspect ratio of the nanorods [23,61]. Particularly for plasmonic NPs, Lee et al. found that silica-coated Au nanorods showed a higher brain accumulation than those with the spherical shape for up to 24 h of time points [24]. 

Considering the unique property of magneto-plasmonic NPs, the external stimulation also plays a significant role in determining their BBB-passage efficiency, as illustrated in Figure 4 and summarized in Table 1. Kong et al. found that the application of an external MF can help silica-coated SPIO NPs to cross the BBB and target a specific brain area through a mechanism of transcellular trafficking [51]. Huang et al. found that the existence of an external MF can facilitate the active penetration through the BBB and enhance the accumulation of tween-80 modified SPIO NPs in the frontal cortex of the brain by two and a half times [52]. Our recent work built a physiologically based pharmacokinetic (PBPK) model to simulate the BBB-passage behavior of PEG-modified SPIO–Au NPs, and proved experimentally, theoretically, and numerically that a static MF enhanced the BBB crossing of PEG-modified SPIO–Au NPs in rats [53]. However, one of the problems for SPIO–Au NPs is that the magnetic-targeting efficiency is still low because of the small size of the SPIO core and large portion of Au, which restricts their further application in drug delivery and neuroregenerative modulation. In a recent work, Bornacelli et al. reported the ferromagnetic properties of plasmonic Pt NPs by delimiting their size to 2–5 nm [62]. In their work, a photomagnetization phenomenon was also observed under the picosecond-pulsed light excitation of ultrasmall Pt NPs. These type of small plasmonic metal NPs, which exhibit ferromagnetic properties, can overcome the limitation of the current SPIO–Au NPs by enabling the much simpler sample-synthesis process, which reduces the mass of a single NP and increases the magnetization rate. Additionally, because of the high spatial and temporal resolution of light [63], the photomagnetization effect of Pt NPs also suggests their possible application in neuronal regeneration when the precise neuronal modulation at the targeted site is required. 

Light, as another type of external stimulation, can also affect the BBB permeability. Li et al. reported that the transcranial 532 nm picosecond-laser excitation of plasmonic Au NPs targeting the tight junction can temporary increase the BBB permeability by enhancing the diffusion through the tight junction in vivo [54]. Additionally, focused ultrasound (FUS) is another demonstrated noninvasive stimulation technique that has been reported by clinical trials [55] to enable the BBB opening by the FUS-induced oscillation of microbubbles (MBs) near endothelial cells, which leads to the temporarily disrupted junctional complexes and allows the delivery of therapeutic agents by passing through the BBB [64]. For example, Chan et al. reported that the use of FUS can enhance the delivery of DNA-coated Au NPs to a specific brain area of mice [56]. Furthermore, magneto-plasmonic NPs can be used with combined FUS and MF to enhance their BBB-passage capacities. It is reported that combined FUS-and-MF stimulation enables the enhanced delivery efficiency of plasmid DNA (pDNA) by polyethylenimine (PEI)-coated SPIO coupled MBs [55]. This effective gene delivery led to a 3.2-fold increase rate in the dopaminergic-neuron recovery in a mouse model of PD by boosting the transfection rate of the gene through the enhanced permeabilization of the cell membrane, and inducing the cytoskeleton reorganization by MF [55]. The magnetic force exerted on the SPIO NPs strongly increased the local pDNA concentration and enhanced the pDNA accumulation in the cellular nucleus. Importantly, the authors developed a two-step stimulation strategy: the first step is the MF + FUS (1 MHz) stimulation, including the magnetic navigation of SPIO NP coupled MBs to reach the brain and the opening of the BBB by FUS, which is followed by the second stimulation of the MF after 24 h to promote cytoskeleton reorganization after pDNA-loaded SPIO NPs enter into the cells and escape from the lysosome. This work suggested that the different types of external stimulations can be finely programmed to meet the needs of biological reactions and processes at different times and locations in an animal model and human body by enhancing the BBB passage of magneto-plasmonic NPs for brain-tissue regeneration.

Thus, the BBB penetrability of magneto-plasmonic NPs is not determined exclusively by any single factor, but relies on all the parameters, including the size, shape, and surface functionalization, as well as the existence of external stimuli, which need to be considered carefully in the design of NP-based disease-modifying treatments for brain-tissue regeneration.

## 5. Magneto-Plasmonic NPs for Drug Delivery

### 5.1. Glial-Cell-Derived Neurotrophic-Factor (GDNF) Delivery

GDNF is an essential biomolecule in the self-renewal, maintenance, and proliferation of neural cells. This role renders it a potential treatment in NDs—particularly in the treatment of PD. Unfortunately, GDNF has demonstrated little success in clinical trials, despite its perceived potential in the neuroprotection and regeneration of neural cells [65]. There exist possible explanations, which include a reduction in Nuclear-receptor-related 1 (Nurr1) expression [65], the use of GDNFs in late-stage Parkinson’s, where it would not be as effective [66], inaccurate representations of PD in preclinical animal models [67], and poorly designed catheter systems in the intracranial delivery of GDNF [68]. One of the more pertinent issues, however, is the inability of GDNF to cross the BBB without outside stimulation or the use of invasive neurosurgical procedures—the latter being where GDNF delivery has seen more success. Moreover, it is less ethically tolerable in human PD patients due to safety risks associated with intracranial surgery. Here, we explore methods of GDNF delivery that would circumvent the need for invasive surgeries. 

GDNF, having a molecular size of ~24 kDA, is unable to cross the BBB independently to reach targeted areas of the brain [69,70]. This necessitates the use of assistive technologies (FUS, surgery, MF, etc.). One promising solution is the use of GDNF mimetics, with examples including miRNAs miR-182-5p and miR-183-5p, instead of GDNF [71]. GDNF mimetics are smaller than GDNF molecules, and they can stimulate neural cells that express GDNF. While GDNF mimetics have poor aqueous solubility, recent work has aimed to address this through the development of NPs that can improve dissolution [70]. This indicates that further work could go into the development of biocompatible magneto-plasmonic NPs that are able to better support the dissemination and transportation of GDNF mimetics into the brain using sources of external stimulation, such as MFs. 

**Figure 5:**
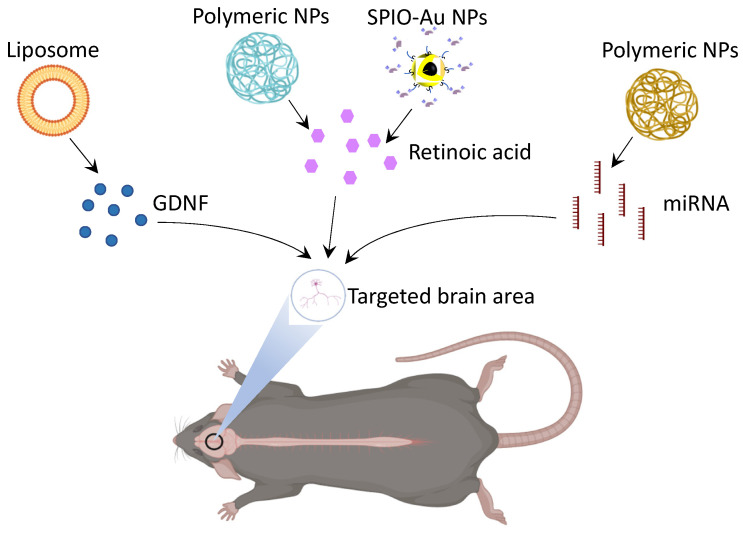
Schematic illustration of drugs and associated drug-delivery systems as potential treatments for ND. (Created with BioRender.com on 18 April 2022).

In the delivery of GDNF using NPs, there has been work on the development of a mechanism for the noninvasive delivery of GDNF NPs in early stage PD rat models (Table 2). Several groups have intranasally used NPs as a delivery mechanism to the brain, such as gold nanoclusters [72], lipid nanocarriers [73], and plasmid DNA NPs [74]. While successful in delivering GDNF across the BBB, a limitation of the intranasal mode of delivery presented here is that it is nonspecific in its targeting of brain regions, which means the mass dissemination of GDNF in the brain instead of focused on areas affected by early stage PD. This leads to lower concentrations of GDNF at the target site, and it may induce side effects that are often associated with nonspecific GDNF targeting, such as anorexia. Thus, there is a need for a more efficient NP-based method of targeted delivery. Another method of delivering GDNF across the BBB would be the utilization of liposomes (Figure 5). Wu et al. indicated the successful delivery of GDNF across the BBB using sterically stabilized liposomes [75]. Similar to the papers that discuss intranasal delivery, the delivery of GDNF here was also largely nonspecific and noncontrolled.

In summary, the primary challenges in the delivery of GDNF are its size, controlling the drug release, and the targeting of specific brain regions. Nanocarriers have demonstrated the ability to transport GDNF across the BBB [83]; however, there is still a gap in the development of NPs that are capable of increasing the efficiency of GDNF uptake, delivery across the BBB, and site-specific targeting to realize the full potential of GDNF-conjugated NPs in the treatment of early stage PD. The magneto-plasmonic NPs, because of their magnetic/light-stimulable properties, could be a potential candidate for the targeted and controlled delivery of GDNF.

### 5.2. MicroRNA (miRNA) Delivery

Another possible therapeutic in the treatment of ND is the delivery and regulation of miRNAs. miRNAs, in different contexts, can either be supportive in the maintenance of neural health, or an underlying contributor to NDs. Thus, it may be important to investigate the potential delivery of miRNAs and anti-miRNAs as regulators of the gene expression in NDs. Similar to GDNF, the delivery of miRNA regulators into the brain is limited by the presence of the BBB, their poor stability, their potential to induce nonspecific immune responses, and their limited half-lives [84]. However, the use of inorganic magnetic NPs in the delivery of miRNAs, miRNA regulators, and miRNA mimetics demonstrated potential in PD rat models (Table 2) [85]. For example, the delivery of polymeric magnetic NPs loaded with miRNA-targeting oligonucleotides is reported by Titze-de-Almeida et al. to regulate specific miRNA expressions (Figure 5) [76]. They utilized intracerebroventricular injections of the magnetic NPs into the rat brain—just as with GDNF. However, this is not a viable option for NP delivery in human models due to the inherent invasiveness of the procedure [76]. The Niu et al. paper forgoes surgical methods of NP delivery, which would echo the ethical barriers in GDNF studies [77]. They instead injected 1-methyl-4-phenyl-1,2,3,6-tetrahydropyridine to model PD, and intraperitoneally administered the magnetic NPs loaded with shRNA plasmids. They present evidence of these magnetic NPs successfully crossing the BBB, with a significant reduction in Parkinson-like symptoms, and without external stimulation (e.g., MFs, light). Currently, there is still a lack of literature detailing the noninvasive delivery of unmodified miRNAs to specific regions of the brain using magnetic NPs. The different molecules and miRNAs used in the treatment of ND are limited by the presence of the BBB. Thus, it is necessary to develop other noninvasive or less invasive approaches based on magneto-plasmonic NPs to realize the controlled and efficient delivery of these drugs to the desired portions of the brain.

### 5.3. Retinoic Acid (RA)

Hydrophobic drugs, such as RA, have been reported for their therapeutic effect on neuronal survival [78] and differentiation [86]. The administration of RA is usually challenging due to its poor water solubility and short half-life in serum and blood [79,87]. The undesired side effects due to the localized high concentration of RA also need to be minimized [80,88,89]. The encapsulation of RA into NPs can be a potential approach to overcome these existing limitations [90]. For example, liposomes and copolymers [79], polymeric NPs [81,87], and polyethylenimine/dextran sulfate NPs [82] have been used to deliver RA to improve its water solubility and circulation time for neuronal differentiation (Table 2, Figure 5). The delivery of RA through the polymeric NPs induced a neuroprotection effect in a PD mouse model by reducing the loss of dopaminergic neurons and increasing the expression levels of Pituitary homeobox 3 (Pitx3) and Nurr1, as transcription factors related to dopaminergic neuronal survival and growth [78]. The surface of the nanocarrier can be functionalized and modified to extend the half-life of RA and improve the BBB-targeting ability. However, these existing nanocarriers lack the function to enable controlled and sustained RA release. In our previous work, we reported a novel smart nanocarrier system by conjugating SPIO–Au core–shell NPs with porous coordination cages (PCCs) (Figure 5) [2]. In this system, the external low-intensity light-emitting diode (LED) was used to control and accelerate the release of RA, which was about 100% release, compared with 40% in the control group, which was not light-stimulated. This study demonstrated the effect of low-intensity light combined with magneto-plasmonic NPs to realize the controlled release of RA, and it also suggested the potential use of multifunctional SPIO–Au NPs for controlling the release of other similar hydrophobic drugs at a desired rate using external-light stimuli.

### 5.4. Current Challenges of NP-Based Drug Delivery

Although the encapsulation of therapeutic drugs by NPs enhanced the BBB permeability, the circulation time, and the therapeutic effect, there are still restrictions in the current approaches, including the risk of too-high concentrations of drugs and NPs in some brain areas, the limited transportation of drugs to the desired areas of brain, and the too-fast circulation time of some NPs, which requires the frequent administration of nanocarriers and may induce some side effects. One of the possible solutions is to combine magneto-plasmonic NPs with external stimulation to enable a controlled and sustained drug-release pattern. Therefore, a further extended drug-release time window can be achieved to maintain the effective drug concentration during a certain time and minimize the number of dosages to reach the effective drug level. Another challenge is that only a small portion of NPs can reach the brain. There is a rising need to develop technologies to increase the number of NPs that can be transported into the brain area, and to reduce the accumulation of NPs in other organs and tissues. A promising way is to use magnetic NPs as drug carriers, which can be noninvasively directed by MFs towards a specific brain area.

## 6. Stimulation-Controlled Drug Release/Therapy: Potential for Brain Regeneration

NPs have been widely used as drug carriers. However, not all these can achieve the goal of controlled delivery. To control the release of the drug from nanocarriers, certain stimuli, which can activate the release process, are needed. The sources of stimuli can be divided into two categories: endogenous and exogenous [91]. 

Endogenous stimuli usually take place in vivo and are controlled by the pH level, redox potential, and presence of certain ions or enzymes. These specific conditions, which normally only exist in certain lesion areas, such as tumor or inflammation tissues, can trigger the release of the drug on site [92]. Drug-delivery systems based on endogenous stimuli are suitable for certain applications, such as tumor treatment. The limitation is that they will lose the control ability when the application comes to other tissue areas, such as neuroregeneration.

Exogenous stimuli are physical stimuli that can be applied to the targeted area from the outside, and they include temperature, light, FUS, and MFs [93]. Exogenous stimuli have attracted interest due to their noninvasive/minimally invasive nature. First, most exogenous stimuli can be applied remotely, without direct invasion into the targeted area. This is desired for routine treatments. Second, the release of drugs from carriers can be controlled on demand, which can prevent the common burst-release problem of traditional drug-delivery systems [94]. The drug carriers need to be designed as or integrated with functional materials that interact with such physical stimuli. For example, to realize light-controlled release, light-sensitive polymers, or NPs with photothermal effects, are required. The same rule applies to other stimuli. The following discussion gives a general summary of external-stimuli-controlled drug release (Table 3).

### 6.1. Light Stimuli for Controlled Drug Release

Light stimuli mainly promote the drug release by photochemical and photothermal mechanisms. In the photochemical process, light can trigger the structure transformation or degradation of certain materials, and, in the photothermal process, the light can be absorbed to lead to a rise in the local temperature. The photothermal effect is based on the plasmonic materials that can absorb light and convert the photons’ energy into heat. Noble-metal NPs have strong LSPR, which can lead to high-efficient photothermal conversion [101]. Among these, Au NPs are gaining a lot of interest due their great optical properties [102], good biocompatibility [33], and ease of functionalization [103]. 

When designing light-responsive drug carriers, it is important to take the light-penetration ability into consideration, as the light stimuli need to go through skin, tissues, and even the skull before reaching the targeted organ. The light-penetration ability is highly dependent on the range of the absorption wavelengths of different biological tissues. Considering the energy loss caused by absorption by blood, two common optical windows are from 650 to 950 nm and from 1000 to 1350 nm [104]. This desired wavelength range is a special requirement for the nanocarriers, as their LSPR wavelength is determined by their shape and size. Based on the theoretical calculation, the LSPR wavelengths of different Au-based NPs are predicted [105]: First, different morphologies of Au NPs have very different wavelength ranges (i.e., solid gold nanospheres have their LSPR in the green-light range, while gold nanoshells and gold nanorods are mostly in the NIR range). Second, with the same morphology, the LSPR wavelength will redshift with the increase in the size (radius (r) for spheres and shells, or effective radius (r_eff_) for nanorods); thirdly, for nonspheric NPs, the larger the core–shell ratio (r_in_/r_out_) (for nanoshells) or aspect ratio (R) (for nanorods), the longer the wavelength will be. 

**Figure 6:**
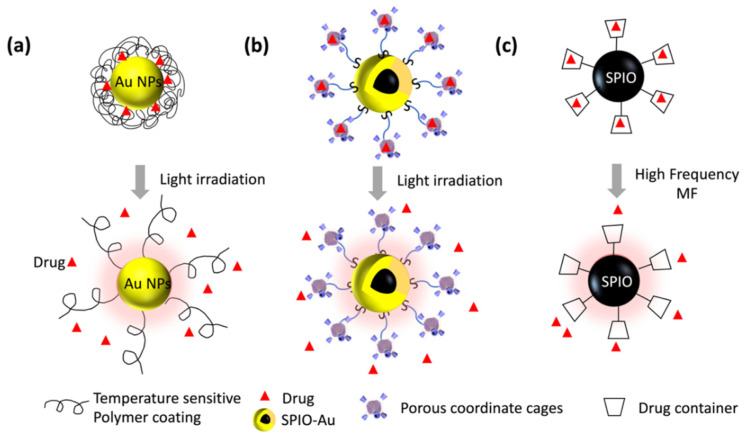
Schematic of different stimulus-type-triggered drug release: (**a**) light-stimulated drug release from Au NPs and drug-encapsulated temperature-sensitive polymers via plasmonically generated heat; (**b**) light-stimulated drug release from PCC-conjugated SPIO–Au NPs via plasmonically generated heat; (**c**) high-frequency MF-triggered drug release from drug container conjugated with SPIO NPs via hyperthermia effect.

There are several reported works that use Au NPs as light-controlled drug carriers. Nanoporous Au NPs [95] and mesoporous Au NPs [96] have been shown to realize the controlled release of doxorubicin under the irradiation of light. It is also reported that Au NPs combined with temperature-sensitive hydrogel can realize the “on” and “off” drug release using 532 nm light stimulation [97]. In this work, the light-to-heat conversion by Au NPs could accelerate the phase transition of the hydrogels, which, in turn, triggered the drug release, as shown in Figure 6a. Particularly for brain disease, the RA-loaded light-responsive NPs exhibited a triggered release of RA under the irradiation of 405 nm of blue light [106]. The blue light also helps in transiently increasing the reactive oxygen species, which leads to the increased RA receptor levels. Therefore, the combined stimulation of light and NPs is capable of amplifying the neurogenesis effect in vivo and in vitro, and of realizing the controlled release of RA. Light at 550 nm was also shown to activate the mitochondrion-targeted delta-rhodopsin and, therefore, to protect the mitochondrial functions in a drosophila model of PD [107]. Our recent work reported a controlled RA-release system that used a 525 nm LED light to stimulate SPIO–Au NPs functionalized with porous coordinate cages (PCCs). The plasmonic heat generated on the SPIO–Au NPs enhanced the release of RA from the PCCs, compared with the control groups, which had no light stimulation, as shown in Figure 6b. However, there is still limited work that uses light to realize the controlled delivery of other therapeutic drugs for brain disease. It is expected that, by programming the light stimulations via the complicated setting of parameters, such as the light intensities, durations, and NP dosages, the precise control of light–NP interactions, and their functions on neurons, can be realized.

### 6.2. MF Stimuli for Controlled Drug Release

Magnetic NPs have been used for bioimaging and the targeted transportation of drug carriers [108]. Moreover, applying the time-variant MF (or alternative MF (AMF)) can induce a temperature increase in magnetic NPs. The principle of this concept is that the magnetic NPs will realign their magnetic moment with the direction of the external field, and will dissipate energy as heat during this process [98]. To release the controlled delivery with AMF, special materials that are responsive to the heat and AMF are needed. These can be magnetic NPs and thermally sensitive materials, or other materials with temperature-sensitive bonds. SPIO is widely used as such a type of material. It can be integrated with drug-loading materials through surface decoration, as illustrated in Figure 6c, or just simply wrapped into the drug carrier.

The work reported by Yogo et al. decorated the surface of SPIO with both β-Cyclodextrin (CD) for drug loading, and folic acid (FA) for tumor targeting [99]. As a common supramolecular host molecule, the CD is featured with a hydrophobic inner cavity and outside hydrophilicity, which provided the loading capacity of tamoxifen (TMX) (an anticancer drug) in this case. The application of an AMF of 230 kHz in frequency can raise the temperature from 37.0 °C to 42.5 °C in less than one minute. Such a temperature increase accelerates the release of the drug, which enables the controlled release by the on–off switching of the MF.

Another case is to assemble layer-by-layer microcapsules with embedded magnetic NPs [98]. Contrary to other reported work, this type of drug carrier does not require a high frequency to generate much heat to trigger the release. Instead, by applying a low-frequency (50 Hz) AMF, the magnetic NPs in the polymer shell will twist and vibrate, which leads to the distortion of the multilayers, which thus increases the permeability of the microcapsule. The controlled and sustained release of DOX is successful, as the capsule’s structure is not broken, which avoids the common burst-release problem. However, the difference in the drug-release rate between the AMF group and the controlled group is not obvious, which is possibly due to the low thermal effect.

Recently, a strategy of remote chemomagnetic modulation for neural circuits was reported using thermal-responsive magnetic NP-loaded liposomes as the drug carrier of a chemical neuromodulator [100]. The structure of the liposomes was constructed by amphipathic lipids, which created a hollow spheric shell containing the inner hydrophilic void space filled with water, and the hydrophobic lipid bilayer. The liposome’s permeability will rapidly increase when the temperature rises to its phase-transition temperature. In this case, the frequency of the AMF was optimized to 150 kHz to achieve a good drug-release performance, while maintaining the structural integrity. The controllable drug release by the phase transition of liposomes is triggered by AMF-induced hyperthermia. This chemomagnetic approach enables the ligand–receptor regulations. However, there is still limited work that uses AMFs combined with magneto-plasmonic NPs to realize the controlled delivery of other therapeutic drugs for the modulation of the neuronal function. The AMF-triggered thermal effects by magneto-plasmonic NPs also have strong potential to combine with other endogenous or exogenous stimuli to realize the programmable drug delivery for neuron regeneration. 

### 6.3. Magneto-Plasmonic Effects for Brain-Tissue and Cellular Regeneration

**Figure 7:**
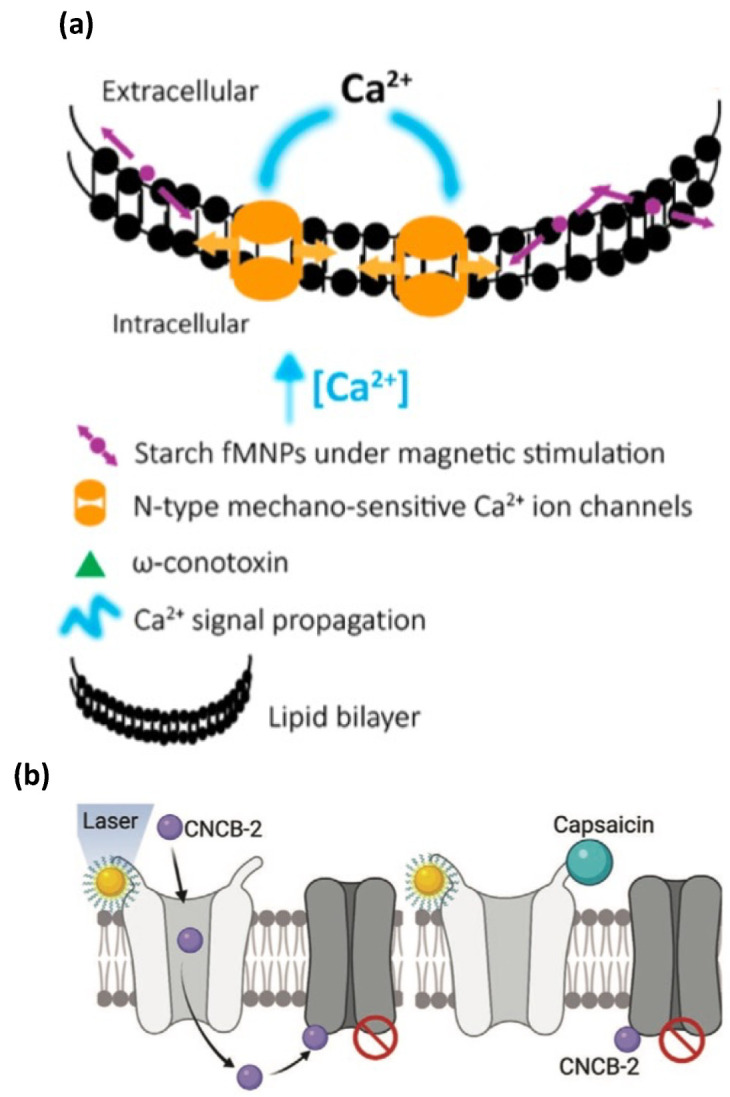
Graphical illustration of the mechanism of magnetic and plasmonic stimulation on neurons: (**a**) magnetic stimulation on magnetic NPs, which are located at cell membrane, to open mechanosensitive calcium channels (Copyright 2016 by ACS); (**b**) plasmonic stimulation of gold NPs to activate the opening of TRPV1 channel, and then allow the entrance of an N-type calcium-channel blocker (Copyright 2022 by Wiley).

The magneto-plasmonic effects via MF/light-stimulated NPs exhibit different functions in affecting neuronal activity and cellular regeneration. On the one hand, the magnetic effects are reported to enhance the mitochondrial function [109], trigger the opening of mechanosensitive ion channels (Figure 7a) [110] and thermally sensitive ion channels [111], activate the neuronal genes [112], etc. For example, Umarao et al. [109] found the beneficial impact of SPIO NPs (20 nm) exposed to an electromagnetic field (EMF) (2 h/day, 17.96 uT, 50 Hz) in a 6-OHDA induced rat model of PD (3 ug/2 uL NP direct striatum injection) by enhancing the mitochondrial function and attenuating the lesion volume. It is believed that the nanomagnetic force exerted by SPIO NPs under an external magnetic field leads to the mechanical opening of N-type mechanosensitive Ca^2+^ channels located at the cell membrane [110], and it therefore can be used to remotely control the calcium level in the neuronal network [113]. Others found that transiently electromagnetized Au NPs (35.6 ± 8.8 nm, 16.2 ± 5.2 mV, 2 × 10–3 T/100 Hz) can help to transfer the EMF energy from NPs to cells, and enable a lineage reprogramming to induce dopamine neurons from somatic fibroblasts in vitro and in vivo by activating the histone acetyltransferase Brd2 and leading the acetylation of H3K27 and the activation of neuronal genes. It is important to notice that the reprogramming efficiency can be improved by choosing the specific frequency and intensity, which indicates the programmable potential of electromagnetic stimulation for restoring the function of the dopaminergic neuron in PD [112]. To realize the precise control of neuronal activities via magnetic stimulation, a theoretical stimulation is preferred to save the time and expense of developing brain-stimulation strategies. Le et al. performed a theoretical analysis of magnetic stimulation via the magnetothermal effects of opening the temperature-sensitive ion channels by magnetic NPs [111]. By running the COMSOL-software-based simulation, the basic parameters, such as the volume and concentration of magnetic NPs, the time and frequency of the MF, and the specific loss power of magnetic NPs, can be analyzed according to the bioheat equation. 

On the other hand, the plasmonic effects are reported to inhibit neuronal activities [114], activate heat-sensitive ion channels [115] and light-sensitive ion channels [116], and stimulate action potentials [117]. For example, Jang et al. reported the usage of gold nanorod to absorb NIR light and realize a photothermal neuronal modulation by inhibiting neural activities, such as reducing the spike rate [114]. Others found that the laser stimulation of gold NPs could activate the heat-sensitive transient receptor potential vanilloid 1 (TRPV1) channel in nociceptive neurons, and turn that channel into a drug-entering site to allow the entrance of an N-type calcium-channel blocker for the purpose of silencing targeted nociceptors (Figure 7b) [115]. A similar activation of TRPV1 ion channels via plasmonically stimulated gold nanorods was reported in HEK293T cells [118]. Damnjanovic et al. reported that a precise neural excitation could be realized by utilizing plasmonically stimulated gold NPs coated to a nanoeletrode, which was placed alongside the trigeminal neuron. The action potential was thus triggered by the conjunction of electrical stimuli and light stimuli [117]. The different functions exerted by the plasmonic and magnetic effects suggested that, by programming the magnetic and light stimulations via the regulation of parameters such as the magnetic field parameters, light intensities, durations, and NP dosage, the precise control of NP–stimuli interactions, and their functions on neurons, can be realized.

### 6.4. Other Functionalities of Magenetoplasmonic NPs for Neuroregeneration

Because of the outstanding magnetic and light properties, magneto-plasmonic NPs also possess other functionalities to assist the neuroregeneration process. On the one hand, magnetic NPs have been used as excellent bioimaging contrast agents for MRI-assisted diagnosis [119], and specifically for the diagnoses of brain diseases, such as PD and Huntingdon’s disease [120]. Magnetic NPs could also be used to enhance the targeted migration of the transplanted stem cells into the injured region in a rat model of PD driven by external MFs [121]. It was further shown that silica-coated iron oxide NPs (49.9 nm) could be translocated from the cytoplasm into the neurites in midbrain dopaminergic neurons directed by an external MF [122]. On the other hand, plasmonic NPs have also attracted considerable attention for neuroregeneration. For example, Au NPs are promising contrast agents in computerized tomography due to their high absorption coefficient [123]. The unique optical property further enables the applications of Au NPs in ultrasensitive biosensing probes according to the LSPR-absorbance-band change [124] for the detection of dopamine [125,126] and alpha synuclein [127]. In addition to the application in the biosensor, Au NPs have been widely used in combination with a scaffold to tune the conductivity and electrical activity of cells [128], and to improve the mechanical property of the scaffold [129]. The promotional effect of Au NPs on the maturation and differentiation of stem cells has also been proven [130].

### 6.5. Potential of NP-Enabled Programmable Drug Delivery for Brain Regeneration

Programmable drug delivery by nanocarriers has been explored in cancer therapy, in which it has exhibited notable benefits, including the accurate spatiotemporal control of the drug release, significant increase in the drug efficacy and specificity, and reduction in side effects [131]. For brain regeneration, this strategy also possesses the high potential to improve the bioavailability, realize the controllable site-specific delivery and release of therapeutic agents, and reduce their therapeutic doses for neurodegenerative diseases. To achieve programmable drug delivery, nanocarriers can be constructed with several stimuli-responsive moieties so that the programmed drug delivery and release can be triggered by multiple external stimuli, such as MF, light, temperature, and FUS, as well as internal stimuli, such as pH and enzymatic activities. For example, the programmable drug release can be controlled by two logical gates, which are enabled by four interrelated moieties that construct the nanovesicles: the first gate requires mild hyperthermia and an acidic pH to generate heat for nanovesicle degradation in the tumor microenvironment (acidic condition); the second gate requires the activation of temperature-sensitive components to generate free radicals and the radical-inducible degradation of the nanovesicles for the drug release [132]. Nucleic acid nanocapsules can also be programmed via enzyme-triggered degradation by incorporating the photo-cross-linking peptide into the nanocapsules [133]. Similarly, for the treatment of ND, the programmable drug delivery can be achieved by taking the benefits of multiple stimuli in combination with multifunctional magneto-plasmonic NPs, such as light + MF, MF + FUS, light + FUS, etc. For this purpose, the prediction of the nanocarrier behavior under multiple stimuli effects is of top priority for programmable drug delivery. The traditional brain-stimulation strategies that have been developed so far mainly rely on multiple experimental attempts or numerous simulations to determine the significant parameters of the external stimuli to regulate the neuronal activities, which are usually time-consuming, require high expenses and efforts, and even experience lots of failures. To address this, an efficient scheme is proposed that is called inverse design, which is based on the deep-learning algorithm. The deep-learning-assisted inverse-design strategy enables the mapping between the critical information of the stimuli and NPs and the neuronal-specific parameters, such as the ion levels, and the gene and protein expressions extracted from experimental measurements. It has been used for the prediction and design of the structures of nanophotonic materials. For example, So et al. performed the simultaneous inverse design of the structure-parameter and materials information for core–shell photonic NPs [134]. An artificial neural network was trained to learn the material structures–optical properties correlation and enable the inverse design of the complex photonic materials. A machine learning method-assisted inverse design based on the deep neural network also enabled the mapping between the dimensional parameters of plasmonic NPs and their far-field and near-field optical properties, and the inverse prediction of the critical parameters of the NPs [135]. Compared with the traditional parameter-optimization method, deep-learning-enabled inverse design consumes much less time and provides much higher efficiency to predict the properties and functions of NPs under external-light and magnetic stimulation. This method holds the strong potential of programmable brain stimulation to precisely regulate the neuronal activities. It also allows us to explore and predict the neuronal effects of multiple stimuli in combination with multifunctional magneto-plasmonic NPs by establishing a mapping between the critical information of the stimuli and NPs and neuronal-specific parameters, such as the ion levels, and the gene and protein expressions extracted from experimental measurements. Advances from deep learning and artificial intelligence will provide support for programmable brain stimulation, which is a potentially revolutionary approach for the therapeutic developments for ND. 

## 7. Magneto-Plasmonic NPs in Brain Organoids

**Figure 8:**
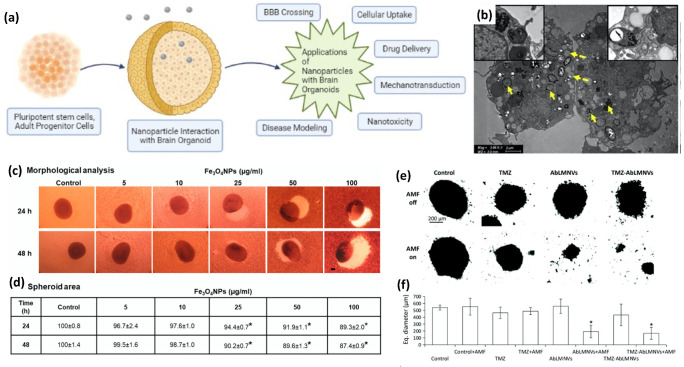
The formation and potential applications of brain organoids to analyze NP effects for neuroregeneration. (**a**) Schematic of brain-organoid formation and potential NP interactions to facilitate development of therapeutic nanomedicines for neuroregeneration (created with BioRender.com); (**b**) AuNP uptake in 5-cell brain organoids inside microglia (yellow arrows) and surrounding endosomes and lysosomes (Copyright 2022 by Elsevier); (**c**) morphological and (**d**) area estimation of human-umbilical-cord mesenchymal stem cell-derived brain organoids after 24 and 48 h exposures to iron oxide NPs (5–100 µg/mL) (Copyright 2022 by John Wiley and Sons); (**e**) transmitted light images and (**f**) size analysis of glioblastoma organoids after 4 days of AMF treatment demonstrating the disintegrative effect of AbLMNVs and TMZ-AbLMNVs. * *p* < 0.05 was statistically considered significant difference. (Copyright 2022 by RSC).

Organoids are self-organizing 3D in vitro culture systems that are derived from either pluripotent or adult stem/progenitor cells to create miniaturized organs. Current 2D cell cultures cannot mimic complex in vivo cellular interactions, while animal models do not accurately represent human effects due to physiological and species differences. This makes human-brain organoids a promising approach to analyze the NP effects on normal and disease states in an ethical and cost-effective manner (Figure 8a). Therefore, brain organoids are a valuable tool for understanding tissue interactions with magneto-plasmonic NPs, and they can further accelerate the development of related therapeutic nanomedicines for neuroregeneration.

### 7.1. Cellular Uptake of NPs in Brain Organoids

In brain organoids, the outer layer can be utilized to simulate the BBB, which prevents the transmigration of drugs or particles from the cell culture medium into the core. Human-brain endothelial cells, pericytes, and astrocytes under low adhesion can spontaneously form an organoid and self-assemble into a structure that resembles the BBB [136]. With this model, each cell type is able to interact with one another and plays an essential role in the maintenance of the BBB integrity and function [137]. In fact, Sokolova et al. used this approach to assess the uptake of Au NPs (2 nm) in a brain organoid consisting of a core composed mainly of astrocytes, covered in pericytes, and with brain endothelial cells forming the surface layer [138]. The authors functionalized Au NPs using click chemistry to attach fluorescein and cyanine-3 fluorescent markers, in addition to functionalization, with the synthetic fluorophore-labeled peptide CGGpTPAAK-5,6-carboxyfluorescein. All three functionalized Au NPs were able to cross the vascular barrier and enter the organoid, while the dissolved dye molecules without Au NPs could not. This showed that a soluble drug can be attached to Au NPs for transportation across the BBB. In addition, Leite et al. analyzed the uptake of Au NPs functionalized with sodium citrate (17.5 nm) and PEG (5.4 nm) in brain spheres derived from human-induced pluripotent stem cells [139]. The authors found that there was cellular uptake within the cells in the brain sphere, with no effect on the viability after 24 h for both types of functionalized Au NPs, and with the PEG–Au NPs having a greater uptake. More recently, Kumarasamy and Sosnik developed a more sophisticated brain organoid that consists of five cell types, including microvascular endothelial cells, pericytes, astrocytes, cortical neurons, and microglia, to assess the endocytosis of Au NPs (10 nm) across the BBB [140]. The authors found that the Au NPs crossed the BBB and were distributed in the cytosol and inside the endosomes and lysosomes of the microglia (Figure 8b). Taken altogether, brain organoids can be a valuable tool to assess the BBB crossing of NPs in a physiologically relevant human model. 

### 7.2. Nanotoxicity Evaluation in Brain Organoids

Currently, 2D monocultures are the most utilized in vitro method to analyze nanotoxicity, but the lack of 3D structures and cellular cross-talking limits their applications in vivo. Organoids provide advantages to 2D monocultures by having relatively long lives, and they can mimic the structural, functional, and genotypic properties of respective organs [141]. In addition, brain organoids can provide more physiologically relevant analysis because they can be derived from human cells so that the interspecies differences in drug metabolism can be circumvented [142]. Thus, the utilization of brain organoids to evaluate NP toxicity can further enhance the development of nanomedicines in the future. 

To evaluate the impact of iron oxide-NP-induced neurotoxicity, De Simone et al. developed multiple brain organoids derived from human D384 astrocytes, SH-SY5Y neuronal-like cells, and human-umbilical-cord mesenchymal stem cells [143,144]. The short-term exposure to iron oxide NPs resulted in differential cytotoxicity in astrocytes and neurons at concentrations of 10 µg/mL and 25 µg/mL, respectively. However, the long-term repeated exposure of iron oxide NPs to these brain organoids after 30 days resulted in time-dependent cell mortality at 10 µg/mL for astrocytes, and 0.5 µg/mL for neurons, which indicates that neurons are more susceptible to iron oxide NPs than astrocytes. In the brain organoids derived from human-umbilical-cord mesenchymal stem cells, the authors found a concentration-dependent decrease (20–60%) in the viability after 24 h and 48 h of exposure to iron oxide NPs in a concentration range of 5–100 µg/mL with 100 µg/mL, which resulted in the greatest mortality and reduction in the organoid area (Figure 8c,d). This decrease in the viability was corroborated by a decrease in the ATP content in the brain organoids. In addition, the neuronal markers, enolase, β3-Tubulin, and microtubule-associated protein 2, were downregulated after 48 h of exposure to iron oxide NPs at a concentration of 50 µg/mL. Based on these brain-organoid models, it is clear that NP-induced neurotoxicity is differentially regulated by cell types, which further demonstrates the need to develop sophisticated organoid models to better predict NP effects in vivo. 

### 7.3. Potential Magneto-Plasmonic-NP Treatments in Brain-Organoid Disease States

Brain organoids cannot only be used to model healthy brains, but also brain diseases. In fact, Chiang et al. utilized a 3D bioprinter to formulate a brain organoid derived from human neural stem cells exposed to amyloid-beta (Aβ) to evaluate the beneficial effects of Au NPs in an Alzheimer’s Disease (AD) model [145]. The application of Au NPs improved the viability of human neural stem cells exposed to Aβ, reduced the expression of inflammatory cytokines, and rescued the levels of the transcripts of inhibitory kappa B kinase. In addition, the Au NP treatment protected the neural stem cells from oxidative stress, and the Aβ-induced reduction in the nuclear factor erythroid 2-related factor 2 expression and downstream antioxidant target genes. This brain-organoid model provided valuable molecular-mechanism insight into the effect of Au NPs in an AD model to facilitate the development of novel treatments. 

In addition to AD brain organoids, glioblastoma organoids have been developed to study NP interactions in brain cancers. Wu et al. developed a surfactant-free ferrofluid containing SPIO NPs (SPIONs) coated with silicate mesolayers and carbon shells (SPION/SiO_2_/C) to analyze the cytotoxic effect on glioblastomas [146]. The authors evaluated the cytotoxicity of SPION/SiO_2_/C on the 2D cultures and 3D organoids of E297 human glioblastoma cells and, after 48 h of exposure, the cell viability was decreased in the 2D monolayers, but not in the 3D organoids, which shows that flat monolayer models cannot be translated into solid tumors. However, the authors found that the SPION/SiO_2_/C restricted the ability of tumor cells to migrate, which can limit the ability of the cells to metastasize to other tissues. A more sophisticated glioblastoma-organoid model was developed by Marino et al., which consisted of a multicellular model of the BBB using brain endothelial cells and astrocytes, and separating a luminal from an abluminal compartment, which contained the cancer organoids [147]. This allowed the authors to study the BBB crossing to target glioblastoma organoids using SPIONs and temozolomide-loaded lipid magnetic nanovectors (TMZ-AbLMNVs) functionalized with an antibody against the transferrin receptor. To facilitate the transport of the TMZ-AbLMNVs, a static MF (2.9 kg of attraction force) was placed below the abluminal compartment to direct the TMZ-AbLMNVs to the glioblastoma organoid. The application of a static MF increased the BBB crossing of the TMZ-AbLMNVs, and further enhanced the glioblastoma targeting after the BBB crossing. The authors also utilized AMF (16 mT, 753 kHz) as a cancer treatment once the TMZ-AbLMNVs crossed the BBB and targeted the glioblastomas. After 4 days of AMF treatment, the glioblastomas were disintegrated and the resulting biological material consisted of mainly cell debris, while the cells that were collected consisted of 87.5% necrotic cells, 7.7% healthy cells, and 4.8% apoptotic cells (Figure 8e,f). Using this sophisticated multicellular model, the authors demonstrated the potential of the enhanced crossing and targeting of glioblastomas under a SMF and utilizing AMF treatment to kill cancer cells in a cost-effective manner compared with in vivo models. According to these previous works on the use of brain organoids to analyze tissue interactions with NPs, it has particularly strong potential for demonstrating the neurotoxicity, BBB crossing, and therapeutic potential of magneto-plasmonic-NP-based nanomedicines. 

## 8. Conclusions and Future Prospects

In conclusion, the type, toxicity, and cellular uptake of magneto-plasmonic NPs are summarized. Particularly, the recent advances in how the external stimuli affect the cellular uptake and BBB passage of magneto-plasmonic NPs are reviewed, which is of great significance in determining the efficiency of magneto-plasmonic NPs as therapeutic agents for ND. We also summarized the potential of magneto-plasmonic NPs for targeted delivery and controlled drug release under the stimulation of light and MF. The existing work suggests that the application of magneto-plasmonic NPs could provide benefits to the delivery of new drugs, or as optimized drug carriers by embedding various stimuli-responsive moieties. Thus, the programmable drug release/stimulation as a new therapeutic strategy for ND can be realized based on the strong potential of the structural/surface modification of magneto-plasmonic NPs. In addition, the utilization of brain organoids to analyze the interaction between neuronal tissues and magneto-plasmonic NPs is summarized, which provides a potential 3D mimicking approach to evaluate the cellular response of magneto-plasmonic NPs and, thus, can further assist the development of the programmable drug release/stimulation for ND. 

## Figures and Tables

**Table 1:** Summary of the effects of external stimuli, including MF and focused US, on enhancing cellular uptake and BBB transfer of magneto-plasmonic NPs.

NP Type	Size	Cell Line/Animal Model	Coating/Functionalization	Observed Effects	Stimulation Type	Citations
Iron oxide NPs	13 nm	MeT-5A, L929 and SK-MEL-28 cells	Silica as coating	External MF significantly increased the cellular uptake of SPIONs, which is intensity-dependent.	MF: Ceramic (5.5 T/m) and Nd–Fe–B magnets (38 T/m) for 15 min.	[48]
SPIO@Au NPs	20–25 nm	C6 glioma cells	Gellan gum as coating	External MF improved cellular uptake.	Permanent magnets (0.33 T) for 1.5–4.5 h.	[49]
SPIO–Au NPs	20 nm	Midbrain neurons	Citrate as coating	External MF induced more internalized NPs.	Halbach array MF applicator (−36.19 T/m) for 30 min.	[18]
SPIO–polystyrene NPs	150 nm	10-week-old mice	Silica as coating	MF increased the number of NPs crossing BBB.	Nd–Fe–B magnet implanted, 1000 Oe, 1 h.	[51]
SPIO NPs	11.5 nm	Adult female Sprague−Dawley (SD) rats	Tween-80	External MF enhanced the accumulation of NPs in the cortex, which is near the magnet.	Nb−Fe−B magnet (0.3 T), placed on mouse head, 2 h.	[52]
SPIO–Au NPs	20 nm	C57BL/6 N mice	PEG and Insulin	External MF enhanced the BBB passage of SPIO–Au NPs.	Halbach array MF applicator (1.48 T) at core area, 30 min.	[53]
Au NPs	50 nm	C57BL/6 mice	mPEG and antibody BV11	Transcranial laser stimulation increased BBB permeability.	1 pulse of 532 nm picosecond (ps) laser, applied through skull (25 mJ/cm^2^).	[54]
SPIO–pDNA-loaded microbubbles	2.7 μm microbubble	SH-SY5Y cells, C57BL/6 J mice	PEI coating	Enhanced pDNA delivery by the combination of focused US and MF navigation.	MF navigation for 3 min, and focused US, 1-MHz, 1 min.	[55]
Au NPs	14 nm	Wild-type C57LB/6 mice	Cy5–DNA coating	Focused US enhanced the delivery of Au NPs through BBB.	Focused US, 1 MHz, 250 s.	[56]

**Table 2:** Summary of advantages and disadvantages of current developed drug-delivery systems for the delivery of GDNF, miRNA, and RA for brain disease.

Drug Type	Drug-Carrier Type	Cell Line/Animal Model	Advantages	Disadvantages	Citations
GDNF plasmid	Compacted DNA NPs	6-OHDA model of male SD rats	Intranasal administration is noninvasive and can circumvent BBB.	Lack of targeted delivery and controlled release.	[74]
GDNF	Liposomes	In vitro BBB model and adult SD rats	Liposome can facilitate BBB crossing of GDNF.	Lack of targeted delivery and controlled release.	[75]
miRNA inhibitor	Neuromag^®^ (polymeric magnetic NPs)	Female adult SD rats	miRNA inhibitors can be successfully delivered into striatum.	Invasive, needs stereotaxic surgery to inject NPs, lack of controlled release.	[76]
Short hairpin RNA	Fe_3_O_4_ coated with oleic acid and N-isopropylacrylamide derivative (NIPAm-AA)	MPTP model of PD, male C57bl/6 mice	ShRNA can be released from multifunctional SPIO NPs.	Drug release lacks controllability.	[77]
RA	Polymeric NPs	MPTP model of PD, male C57BL6 mice	Biocompatible nanocarrier, reduce DA neuron loss.	Invasive, lack of targeting.	[78]
RA	Poly(ε-caprolactone)/poly(ethylene glycol)	Brain tumor cell line	Nontoxic and biodegradable, prevent RES attack.	BBB passage is not approved, lack of targeting and control.	[79]
RA	Nanofiber composite	N2A cells	RA release is controlled by loading RA in the core area of the fiber.	Not for BBB passage, lack of targeting.	[80]
RA	Polymeric NPs	SVZ cells, adult male mice, neural stem cells (NSCs)	Can control NSC-cell differentiation, regulate NSC-cell survival, biocompatible.	Lack of targeting and external-stimuli-controlled release.	[81,82]
RA	SPIO–Au NPs functionalized with PCC	PC-12 cells	Realize the controlled drug release under the stimulation of light.	Visible light has low tissue-penetration ability.	[2]

**Table 3:** Mechanisms of different stimulus-type-controlled drug release from magneto-plasmonic NPs.

Drug Type	Drug-Carrier Type	Surface Modification	Cell Line/Animal Model	Stimulation Type	Stimulation Mechanism	Citations
Doxorubicin	Nanoporous Au NPs	Mercaptosuccinic acid and mercaptopropionic acid	Balb/c nude mice, male	Light	Photothermal effect of Au NPs under light stimulation.	[95]
Doxorubicin	Mesoporous Au network	PEG	N/A	Light	Plasmonic heat-controlled drug release via phase-change material.	[96]
Ofloxacin antibiotic	Au NPs	Poly(dimethylacrylamide-co-acrylamide)/poly acrylic acid hydrogel	N/A	light	Plasmonic heat-controlled drug release via phase change of thermoresponsive polymer.	[97]
Doxycycline	Fe_3_O_4_ containing microcapsules	PEI coating	Mouse C2C12 myoblast cells	Low-frequency AMF, 16 mT, 50 Hz.	AMF-enhanced permeability of microcapsule to release drug.	[98]
Tamoxifen	SPIO	Folic acid and β-Cyclodextrin functionalization	Murine microglialcells	High-frequency MF (HFMF), 50–100 Oe, 230 kHz	High-frequency MF-induced hyperthermia depressed hydrophobic interaction and accelerated drug diffusion from NPs.	[99]
Chemical neuromodulators	Iron oxide magnetic NPs	1,2-dipalmitoyl-sn-glycero-3-phosphocholine (DPPC), 1,2-distearoyl-sn-glycero-3-phosphocholine (DSPC)	Male C57BL/6 mice	AMF, *H*_0_ = 45 ± 2 mT *f* = 150 kHz	AMF-induced hyperthermia triggered drug release from thermally sensitive lipid.	[100]

## Data Availability

This is a review article, and the data can be found in the literature.
